# Probing the regulatory effects of specific mutations in three major binding domains of the pleiotropic regulator CcpA of *Bacillus subtilis*

**DOI:** 10.3389/fmicb.2015.01051

**Published:** 2015-10-02

**Authors:** Ruud Detert Oude Weme, Gerald Seidel, Oscar P. Kuipers

**Affiliations:** ^1^Molecular Genetics Group, Groningen Biomolecular Sciences and Biotechnology Institute, University of GroningenGroningen, Netherlands; ^2^Lehrstuhl für Mikrobiologie, Institut für Mikrobiologie, Biochemie und Genetik der Friedrich-Alexander Universität Erlangen-NürnbergErlangen, Germany

**Keywords:** CcpA, CcpA mutants, *Bacillus subtilis*, DNA microarray, transcriptomics

## Abstract

Carbon catabolite control is required for efficient use of available carbon sources to ensure rapid growth of bacteria. CcpA is a global regulator of carbon metabolism in Gram-positive bacteria like *Bacillus subtilis*. In this study the genome-wide gene regulation of a CcpA knockout and three specific CcpA mutants were studied by transcriptome analysis, to further elucidate the function of specific binding sites in CcpA. The following three amino acids were mutated to characterize their function: M17(R) which is involved in DNA binding, T62(H) which is important for the allosteric switch in CcpA upon HPr-Ser46-P binding, and R304(W) which is important for binding of the coeffectors HPr-Ser46-P and fructose-1,6-bisphosphate. The results confirm that CcpA was also involved in gene regulation in the absence of glucose. CcpA-M17R showed a small relief of Carbon Catabolite Control; the CcpA-M17R mutant regulates fewer genes than the CcpA-wt and the palindromicity of the *cre* site is less important for CcpA-M17R. CcpA-T62H was a stronger repressor than CcpA-wt and also acted as a strong repressor in the absence of glucose. CcpA-R304W was shown here to be less dependent on HPr-Ser46-P for its carbon catabolite control activities. The results presented here provide detailed information on alterations in gene regulation for each CcpA-mutant.

## Introduction

Gram-negative and Gram-positive bacteria employ a mechanism called carbon catabolite control (CCC) to use carbon sources in a preferential manner (Stülke and Hillen, 2000; Görke and Stülke, 2008; Fujita, 2009). This mechanism ensures optimal use of the available nutrients and results in a fitness advantage in a natural environment. The Gram-positive bacterium *Bacillus subtilis* uses a global transcriptional regulator, carbon catabolite protein A (CcpA), to employ CCC. Together with the seryl phosphorylated form of the histidine-containing protein, HPr, are the main coeffector for transcriptional regulation of various operons, it is involved in regulation of carbon utilization, overflow metabolism, amino acid anabolism and nitrogen assimilation (Sonenshein, 2007; Deutscher, 2008; Görke and Stülke, 2008; Fujita, 2009). HPr is a phosphocarrier protein from the phosphotransferase system (PTS) transferring phosphoryl groups from its histidine 15 residue to EIIA enabling specific sugar transport by EII complexes. The regulatory function of HPr is initiated when a preferred carbon source like glucose is metabolized and the intracellular concentration of fructose-1,6-bisphosphate (FBP) increases. FBP stimulates the HPr kinase/phosphatase (HPrK/P), which phosphorylates HPr at serine 46 and thereby converting HPr into the CcpA-binding form (Schumacher et al., 2007; Görke and Stülke, 2008; Fujita, 2009). Additionally, HPrSer46-P-CcpA complex formation can be stimulated by glucose-6-phosphate (G6P) and (FBP) (Görke and Stülke, 2008; Fujita, 2009). Moreover, there is a second protein effector: the catabolite responsive HPr (Crh) that binds CcpA at the same site as HPr-Ser46-P when Crh is phosphorylated at serine 46 by HPrK/P (Schumacher et al., 2006). The binding of HPr-Ser46-P to CcpA triggers an allosteric switch in CcpA allowing CcpA to bind its cognate DNA sequences, the *catabolite responsive elements* (*cre*) (Stülke and Hillen, 2000; Deutscher, 2008; Görke and Stülke, 2008). These *cre* sites are semi-palindromic sequences with the following consensus: WTGNNARCGNWWWCAW (R is G or A, W is A or T, and N is any base) (Miwa et al., 2000; Schumacher et al., 2011). After DNA binding CcpA can either act as a repressor, i.e., when the *cre* site is downstream of the promoter, (Carbon Catabolite Repression, CCR) or, in much fewer cases, as an activator, i.e., when the *cre* site is upstream of the promoter, (Carbon Catabolite Activation, CCA). However, there are also exceptions to this rule: the *cre* site of the levanase operon is upstream of the promoter but it is repressed by CcpA (Martin-Verstraete et al., 1995). The expression of 10% of the genes in *B. subtilis* are affected by CcpA when glucose is present in the medium (Fujita, 2009), and the expression of 8% of the genes are affected in the absence of glucose (Moreno et al., 2001).

CcpA belongs to the LacI family (Henkin et al., 1991) and consists of an N-terminal DNA binding domain, and a C-terminal core protein containing the HPr-Ser46-P binding site, an effector binding cleft for G6P and FBP and a dimerization domain (Schumacher et al., 2007, 2011). The crystal structures of *B. subtilis* and *B. megaterium* CcpA-HPr-Ser46-P bound to different *cre* sites and structures of CcpA with FBP and G6P show which amino acids are important for DNA binding, for complex formation, and for coeffector binding (Schumacher et al., 2007, 2011). Studies of point mutations in CcpA, HPr, and Crh have contributed to elucidate the molecular function of several amino acids in the complex (Deutscher et al., 1994; Kuster-Schock et al., 1999; Horstmann et al., 2007; Sprehe et al., 2007). However, differential effects of distinct CcpA point mutations on CCR *in vivo* have also been found. This cannot be explained solely by a comparison of the available structures or interaction analyses because other regulators are also involved in gene regulation of carbon metabolism (e.g. regulon specific regulators such as RbsR).

**Figure 1:**
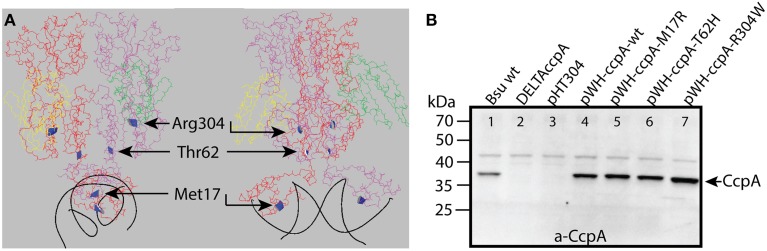
**(A)** The crystal structure of CcpA-HPr-Ser46-P in complex with *the AckA2-cre* site, viewed along the DNA (left) and perpendicular to the DNA (right). The two CcpA monomers were shown in red and purple, the two HPr-Ser46-P monomers were shown in yellow and green, and the DNA was shown in black. The amino acids that were mutated in this study were highlighted in ribbon style in blue (adapted from PDB 3OQM (Schumacher et al., 2011). **(B)** The expression levels of the different CcpA mutants were shown on a Western Blot. Crude extracts of the *B. subtilis* strains with the different *ccpA* mutants were loaded on gel, transferred to a membrane and the CcpA proteins were visualized via chemiluminescence with a CcpA specific antibody. The size of the CcpA protein was 37 kDa. CcpA in lane 4–7 was expressed from plasmid. The *B. subtilis ccpA::spec* strain was utilized in lane 2–7, and complemented with *ccpA* on the indicated pHT304 derived plasmid. The empty pHT304 vector served as negative control.

In this study, we examined the regulons of specific CcpA mutants. Therefore, three specific amino acids in CcpA were mutated (Figure 1A) and examined by transcriptome analyses to study the effects on CCC. Two of these mutants, CcpA-M17R and CcpA-R304W, have been shown in a previous study to differentially regulate *gntR, xynP, alsS*, and *ackA* (Sprehe et al., 2007). Interestingly, these mutants are located in different regions: M17 is in the DNA binding domain and contacts the *cre* site specifically, while R304 makes an important contact to the Ser46-P of HPr. The third mutant, CcpA-T62H was found to repress *xynP* very strongly in the absence of glucose (unpublished data). Threonine 62 is the last residue of the allosteric switch domain, mediating the signal of HPr-Ser46-P binding to the DNA binding domain (Schumacher et al., 2011). The aim of the transcriptome analysis presented here was to study the effect of the pointmutations on a genome-wide level and elucidate the mutant specific regulons. Furthermore we examined the presence of specific correlations between the deregulated or regulated genes and altered *cre* site binding. Will all genes and operons be affected equally by a specific mutation in CcpA or are some genes of the regulon more affected than others? This will provide novel insights on the importance of the residues M17, T62, and R304.

## Materials and methods

### Bacterial strains, plasmids, and oligonucleotides

*B. subtilis 168 trpC2* strains (Table 1) were grown on Lysogeny Broth (LB). *E. coli* MC1061 was used as a cloning host. All plasmids are listed in Table 2 and the oligonucleotides in Table 3. The C-medium contains 70 mM K_2_HPO_4_, 30 mM KH_2_PO_4_, 25 mM (NH_4_)_2_SO_4_, 0.5 mM Mg_2_SO_4_, 10 μM MnSO_4_, 22 mg/l Ferric Ammonium Citrate, 250 μM L-Tryptophan, and 0.4% (w/v) glucose. C-medium supplemented with glutamate contains 0.03% (w/v) L-Glutamate and when it was also supplemented with branched chain amino acids it contained 0.25% (w/v) L-Isoleucine, 0.25% (w/v) L-Leucine, 2.5% (w/v) L-Valine, and 2.5% (w/v) L-Methionine. Uridine 5′-monophosphate (Sigma) was added to C-medium in a final concentration of 20 mg/l.

**Table 1:** ***B. subtilis* strains used in this study**.

**Strain**	**Genotype**	**Source or reference**
*E. coli MC1061*	F^−^*araD139* Δ*(ara-leu)7696 galE15 galK16* Δ*(lac)X74 hsdR2* (${\text{r}}_{\text{K}}^{-}$${\text{m}}_{\text{K}}^{+}$) *mcrA mcrB1 rpsL* (Str^r^)	Laboratory stock, (Casadaban and Cohen, 1980)
***B. SUBTILIS***		
*168*	*trpC2*	Bacillus Genetic Stock Center, (Zeigler et al., 2008)
*DOW41*	*trpC2 ccpA::spec^*r*^*	This study
*DOW42*	*DOW41* with *pHT304*	This study
*DOW43*	*DOW41* with *pWH2422-ccpA-wt*	This study
*DOW44*	*DOW41* with *pWH2422-ccpA-M17R*	This study
*DOW45*	*DOW41* with *pWH2422-ccpA-T62H*	This study
*DOW46*	*DOW41* with *pWH2422-ccpA-R304W*	This study

**Table 2:** **The plasmids used in this study**.

**Plasmid**	**Genotype**	**Source or reference**
pUC18		NCBI accession number L09136
pUC18-Δ*ccpA*	Amp^r^ a*roA Spec*^r^ *ytxD*	This study
pHT304		(Arantes and Lereclus, 1991)
pWH144-*ccpA*-T62H	pHT304 *ccpAhis* T62H	This study
pWH2422-*ccpA*-wt	pHT304 *ccpA*	This study (Arantes and Lereclus, 1991)
pWH2422-*ccpA*-M17R	pHT304 c*cpA-M17R*	This study
pWH2422-*ccpA*-T62H	pHT304 c*cpA-T62H*	This study
pWH2422-*ccpA*-R304W	pHT304 c*cpA-R304W*	This study

**Table 3:** **The oligonucleotides used in this study**.

Oligonucleotide	Sequence (5′ ≥ 3′)
pUC18_FW	AGAGTCGACCUGCAGGCATGCAAGCTTGG
pUC18_REV	ATCCCCGGGTUCCGAGCTCGAATTC
spec_FW	AGCAAGCTUCACCTTTATGGTGAACGTAACGTGACTGGC AAGAG
spec_REV	AAGAAGATUACCAATTAGAATGAATATTTC
*ytxD*_FW	AATCTTCTUGCTTTTTTCATGGGGAGAAATG
*ytxD*_REV	AGGTCGACTCUCTAAGCTTCATGTACAGATCCCTTTTTTG
*aroA*_FW	TACCCGGGGAUAAAAAACCCTTGAACATTG
*aroA*_REV	AAGCTTGCUAACGAGAAAGCATAAAAAAAG
*ccpA*_FW_check	GACGGCATCGTGTTTATGGG
*ccpA*_REV_check	TCTATACGGTGCGGCAGTTC
pUP19	ATTAAGTTGGGTAACGCCAG
pRev19	TCGTATGTTGTGTGGAATTG
*ccpA*mut1	ATAATATCTAGAACCAAGTATACGTTTTCATC
BlpIin	ATAATAATAGCTCAGCTTATGACTTGGTTGACTTTCTAAG
BsuT62rand	ACCTACAGTSNNTGTTTTTTT
hisbam	TATTATTATGGATCCTTAGCTTCCTTAGCTCCTGA

### Recombinant DNA techniques

PCR and DNA purification were done as previously described (Sambrook et al., 1989). Pfux7 DNA polymerase (Norholm, 2010) was a kind gift from Bert Poolman (University of Groningen), USER enzyme was obtained from New England Biolabs. Sequencing was done at MacroGen (Amsterdam, Netherlands).

### Construction of *B. subtilis* CcpA::spec

The *B. subtilis ccpA* knockout strain was made by allelic replacement with a spectinomycin resistance gene. Therefore, 1000 bp flanking regions of *ccpA* were amplified from the *B. subtilis* genome by PCR. The first flanking region was upstream of *ccpA* (primers *aroA*_FW and *aroA*_REV) and the second flanking region was downstream of *ccpA* (primers *ytxD*_FW and *ytxD*_REV). PCR with Pfux7 as polymerase was also used to amplify pUC18 and the spectinomycin gene from pDOW01 (Detert Oude Weme et al., 2015) (see the primers in Table 3). Cloning of the DNA fragments (pUC18, *aroA*, spec^r^, *ytxD*) was done using the uracil-excision DNA engineering method (Norholm, 2010). The ligation product, hereafter called pUC18-Δ*ccpA*, was transformed to *E. coli*. Plasmid sequence was confirmed by sequencing.

Natural competent *B. subtilis* (Harwood and Cutting, 1990) was transformed with pUC18-Δ*ccpA*, which integrated into the native locus of *ccpA*, thereby replacing the *ccpA* gene by a spectinomycin^r^ gene (strain DOW41 in Table 1). In the resulting *ccpA* knockout strain the region from 223 bp upstream till 1037 bp downstream of the *ccpA* translational start site was knocked out; the promoter and the *ccpA* gene were deleted.

Colony PCR and PCR on isolated chromosomal DNA (with primers: spec_FW, spec_REV, *ccpA*_FW_check, *ccpA*_REV_check) were used to check whether the plasmid was inserted via double recombination and whether the *ccpA* gene was removed.

### Construction of the CcpA mutant strains

All previous analyses of the CcpA mutants were done with C-terminally His-tagged CcpA, but the His-tag free plasmid pWH2422 was used here to rule out side-effects resulting from a His-Tag fused to CcpA. This plasmid carries wildtype *ccpA* without a His-tag encoding region under control of its own promoter and a lambda terminator downstream of the gene. For this purpose wildtype *ccpA* was amplified from plasmid pWH144 (Horstmann et al., 2007) using the primer *ccpA*mut1 and BlpIin, restricted with XbaI and BlpI and cloned into pWH144 yielding pWH2422. The plasmid pWH2422-*ccpA*-R304W was cloned analogously with a PCR fragment using the primer *ccpA*mut1 and BlpIin with the template pWH920 (Sprehe et al., 2007) carrying the mutant allele *ccpA*-R304W. Plasmids pWH2422-*ccpA*-M17R and pWH2422-*ccpA*-T62H were subcloned in pWH2422 from pWH1541-*ccpA*-M17R (Sprehe et al., 2007) and pWH144-*ccpA*-T62H via the restriction sites XbaI and ClaI. Plasmid pWH144-*ccpA*-T62H was isolated from a plasmid pool of a randomization of T62. The randomization was done by a two-step PCR mutagenesis using the primers *ccpA*mut1, BsuT62rand, and hisbam.

Natural competent *B. subtilis ccpA::spec* (strain DOW41 in Table 1) cells were transformed with the resulting pWH2422-*ccpA* mutant plasmids as described before (Harwood and Cutting, 1990), and plated on LB-agar plates supplemented with 2 μg/ml erythromycin. Colonies were checked for plasmid integrity with primers pUP19 and pRev19.

### DNA microarray analyses

Lysogeny Broth (LB), supplemented with 2 μg/ml erythromycin when necessary, was inoculated with *B. subtilis 168 trpC2* strains from -80°C and grown overnight at 200 rpm at 37°C. Next morning, the cells were diluted to an OD_600_ of 0.04 in fresh LB with 2 μg/ml erythromycin and 1% (w/v) glucose and grown at 200 rpm and 37°C until the OD_600_ was 0.3. Now 100 ml of cell culture was harvested by centrifugation (6000 rpm, 5 min) and used for RNA isolation. Cell pellets were resuspended in 400 μl TE (DEPC) buffer, and transferred to screw-cap tubes with 0.5 g glass beads (<106 microns, Sigma), 50 μl 10% SDS and 500 μl phenol/chloroform:IAA (a 1:1 pre-made mixture of phenol acid and chloroform:IAA (24:1); from this mixture the organic phase was used). The screw-capped tubes were placed in a bead beater for two times 1 min to lyse the cells. Total RNA was isolated with the High Pure RNA Isolation Kit (Roche). The RNA concentration was measured with the NanoDrop (ND-1000 spectrophotometer, NanoDrop Technologies) and the quality was checked on a 1% agarose gel supplemented with 1% bleach.

Superscript III Reverse Transcriptase (Invitrogen) and 20 μg of total RNA were used for cDNA synthesis as described before (Lulko et al., 2007). Aminoallyl labeled cDNA was labeled with DyLight550 or DyLight650 (Thermoscientific). Now cDNA from *B. subtilis ccpA*-wt cells and cDNA from *B. subtilis ccpA*-mutant cells were mixed in a 1:1 ratio and hybridized overnight on home-made DNA microarray aminosilane glass slides (Lulko et al., 2007). Hybridization of cDNA on home-made slides was done three times with cDNA from three independent experiments, and the third time the dyes were swapped (see the NCBI GEO submission GSE69575 for details).

Slides were washed, dried and scanned with a GenePix 4200AL scanner (Axon Instruments, CA, USA). The images of the scans were analyzed with ArrayPro4.5 (Media Cybernetics Inc., Md, USA).

Further analysis was done with PrePrep (van Hijum et al., 2003), Prep, PostPrep and Cyber-T (van Hijum et al., 2005) as described before (Lulko et al., 2007; Marciniak et al., 2012).

Bayes *p*-values were used to calculate the significance and average spot intensity values were used to calculate the fold change of gene expression as described before (Long et al., 2001; Marciniak et al., 2012). Results shown here are the average of two biological replicates and a technical replicate (dye-swap). Only genes with a Bayes *p*-value smaller than 0.001 and a fold change larger than 1.7 or smaller than -1.7 were used for further analysis.

The microarray data is available at the NCBI GEO database (http://www.ncbi.nlm.nih.gov/geo/) under accession number GSE69575.

### Western blotting

*B. subtilis* cells harboring the plasmids with a *ccpA* mutant were grown as described above. Two milliliters of cells were harvested by centrifugation (5min, 10,000 rpm) at an OD_600_ of 0.3. The pellets were resuspended in 200 μl 50 mM Tris-Cl pH 7.4, a spatula tip of glassbeads (<106 microns, Sigma) was added and subsequently cells were lysed by mini-bead beating (two times 1 min, Mini-Beadbeater-16, Biospec products). After centrifugation (5 min, 10,000 rpm) the supernatant was transferred to a clean tube. Total protein was measured with the DC Protein Assay (BioRad) to ensure that equal amounts of protein were used. 30 μl of 2x SDS loading buffer was added to 20 mg of total protein in a volume of 30 μl and heated for 5 min at 90°C before loading on a 12% SDS-PAGE gel. After electrophoresis, the proteins were transferred to a PVDF membrane (60 min, 80 mA). The PVDF membrane was incubated in PBST + 5% (w/v) skim milk at 4°C overnight. Next morning, the PVDF membrane was washed three times10 min with PBST and subsequently incubated at room temperature for 2 h in PBST + 5% skim milk + 1:10,000 dilution of anti-CcpA antibody (Kuster et al., 1996). The membrane was again washed three times 10 min with PBST and then incubated 1.5 h in PBST + goat-anti-rabbit Ig-horseradish peroxidase (Amersham Biosciences) at room temperature. Now the membrane was washed two times 10 min with PBST. 2 ml of ECL detection reagent (GE Healthcare) and the Molecular Imager ChemiDoc XRS+ (BioRad) were used for signal visualization.

## Results

### Expression levels of the CcpA mutant proteins

The expression levels of the different CcpA proteins were verified by Western blotting with anti-CcpA antibody before starting with the transcriptome analysis. Therefore, cell free extracts from *B. subtilis* harboring the plasmids with the *ccpA* mutants were loaded on a SDS-PAGE gel, transferred to a membrane and incubated with anti-CcpA antibody (Figure 1B). The DC Protein Assay (BioRad) was used to measure the total protein concentration in the cell free extract; this was used to ensure loading equal amounts of total protein on the gel. The *ccpA* on the pWH plasmid was expressed under the control of its native promoter, but despite the low copy number of the plasmid the amount of CcpA was higher than in wildtype *B. subtilis* (Figure 1B). The increase in CcpA expression from this plasmid has been observed before (Sprehe et al., 2007). The expression of the CcpA mutants from plasmid were all very similar, so the observed changes in gene expression in the microarray are most likely due to the mutation in CcpA and not due to changes in the expression level. No CcpA was detected in the *ccpA* knockout strain, indicating the successful removal of the gene (Figure 1B). An unspecific band around 45 kDa was observed for all variants, which was not related to CcpA because it was also visible in the knockout strain and not related to the inserted plasmid because it was also visible in the wildtype strain.

The effects of the CcpA knockout and of the point mutations in the CcpA protein (M17R, T62H, or R304W) were studied on the transcriptome level at the early exponential growth phase (OD_600_ of 0.3) and always compared to the transcriptome of *B. subtilis pWH-ccpA-*wildtype. All strains were grown in LB, either in the presence or absence of 1% glucose. Our goal was to study whether all genes of the regulon will be up- or down-regulated equally or in a differential way. The results will be presented for each mutant separately in the sections below.

### The gene regulation of CcpA knockout compared to wildtype CcpA

The *ccpA* knockout strain had an altered gene regulation for 216 genes when the strain was grown in LB + 1% glucose (**Table 5**, Supplementary File, Sheets 1–3). There is a large variety in the fold changes between the differentially regulated genes, confirming that the strength of transcriptional activation or repression by CcpA differs for each regulon. The expression of 189 of these genes was upregulated, confirming that CcpA mainly functions as a repressor. 61 of these 216 genes were members of the originally annotated CcpA regulon which consists of 213 genes (Mäder et al., 2012; Subtiwiki, 2014). The CcpA regulon as listed in SubtiWiki has been compiled from different papers (personal communication with Jörg Stülke, Göttingen, Germany) and might also contain indirectly regulated genes. Categorizing these 216 genes into COGs showed that most genes were from the groups Energy production and conversion and Carbohydrate transport and metabolism (**Figure 3**). When the *ccpA* knockout strain was grown in LB then the expression of 89 genes was changed (**Table 5**, Supplementary File, Sheets 4–6), of which 31 genes belong to the CcpA regulon as listed in SubtiWiki. 55 genes were more abundant now compared to CcpA-wt, showing that CcpA was still active in the absence of glucose because those 55 genes were repressed by CcpA-wt. Most genes can be categorized again in Energy production and conversion and Carbohydrate transport and metabolism (**Figure 3**). The findings from the *ccpA* knockout strain served as a reference to interpret the results from the CcpA-mutants. Our main questions were: are the CcpA-mutants more or less active in repression or activation than the wildtype? Are all genes affected to similar extents or in a differential way?

### The gene regulation of CcpA-M17R compared to wildtype CcpA

**Figure 2:**
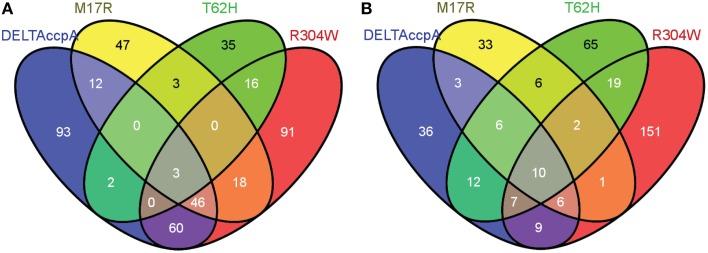
**Venn diagram showing the genes that were differentially regulated in each one of the CcpA mutant strains**. Numbers represent the genes that were differentially regulated in each CcpA mutant strain. Only genes with a fold change larger than 1.7 or smaller than -1.7 were used in the analysis. **(A)** Overview of the differentially regulated genes when the strains with the CcpA mutant were grown on LB + 1% glucose (see also Supplementary File, Sheet 2). **(B)** Overview of the differentially regulated genes when the strains with the CcpA mutant were grown on LB (see also Supplementary File, Sheet 5). Venn diagrams were made via http://bioinfogp.cnb.csic.es/tools/venny/. More detailed information on up- or down-regulated genes is shown in Table 4.

**Figure 3:**
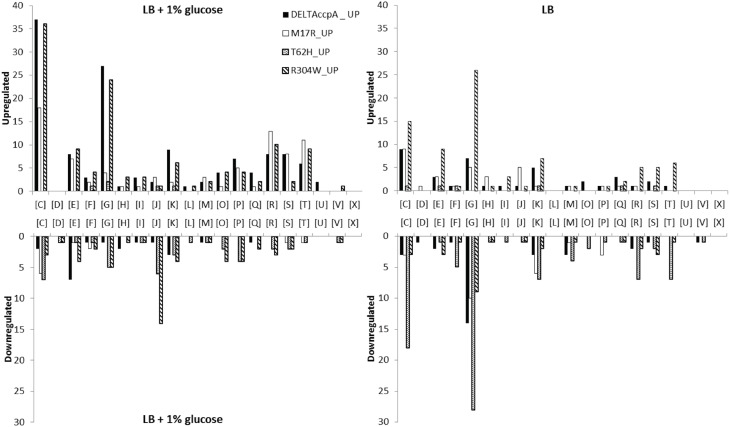
**All differently regulated genes were categorized in Clusters of Orthologous Groups (COGs)**. [C] Energy production and conversion; [D] Cell cycle control, cell division, chromosome partitioning; [E] Amino acid transport and metabolism; [F] Nucleotide transport and metabolism; [G] Carbohydrate transport and metabolism; [H] Coenzyme transport and metabolism; [I] Lipid transport and metabolism; [J] Translation, ribosomal structure and biogenesis; [K] Transcription; [L] Replication, recombination and repair; [M] Cell wall/membrane/envelope biogenesis; [O] Posttranslational modification, protein turnover, chaperones; [P] Inorganic ion transport and metabolism; [Q] Secondary metabolites biosynthesis, transport and catabolism; [R] General function prediction only; [S] Function unknown; [T] Signal transduction mechanisms; [U] Intracellular trafficking, secretion, and vesicular transport; [V] Defense mechanisms; [X] No prediction. Categorization of genes was done on the MolGen server (http://server.molgenrug.nl/index.php/functional-analysis) (see also Supplementary File, Sheets 9, 10).

Most likely CcpA-M17R lost part of its capacity to repress genes, as can be concluded from the large number of derepressed genes. The gene regulation of the CcpA-M17R mutant differed significantly from the ΔCcpA mutant. Of the genes that were affected by CcpA-M17R 53% differed from the genes that were affected in the ΔCcpA strain (Figure 2A and Supplementary File, Sheet 2). The number of affected genes was smaller (**Table 5**) indicating that the function of the CcpA-M17R mutant was similar to the CcpA-wt, but with lowered affinity for most binding sites. When the CcpA-M17R mutant strain was grown in LB + 1% glucose the gene regulation for 129 genes (115 up and 14 down) differed from CcpA-wt (**Table 5**, Supplementary File, Sheets 1–3). Only 27 of these genes belonged to the CcpA regulon from SubtiWiki. Especially genes from COG categories: [C] Energy production and conversion, [R] General function prediction, and [T] Signal transduction mechanisms showed a weaker regulation by CcpA-M17R (Figure 3).

When the CcpA-M17R mutant strain was grown on LB then 67 genes (38 up and 29 down) had a changed gene regulation compared to CcpA-wt (**Table 5**, Supplementary File, Sheets 4–6). Nineteen of these genes belonged to the CcpA regulon from SubtiWiki. Mainly genes from COG categories: [C] Energy production and conversion, [G] Carbohydrate transport and metabolism, and [J] Translation, ribosomal structure and biogenesis showed a weaker regulation by CcpA-M17R (Figure 3). The uniquely affected genes for CcpA-M17R in LB with and without glucose are shown in Table 4.

**Table 4:** **Genes shown here were uniquely up- or downregulated in only one of the CcpA mutants in comparison to CcpA-wt; either in LB + 1% glucose (left side) or in LB without glucose (right side)**.

**LB** + **1% glucose**						**LB without glucose**						
**CcpA-M17R**		**CcpA-T62H**		**CcpA-R304W**		**CcpA-M17R**		**CcpA-T62H**		**CcpA-R304W**		
**47**		**35**		**91**		**33**		**65**		**151**		
**43** **Up:**	**4** **Down:**	**8** **Up:**	**27** **Down:**	**42** **Up:**	**49** **Down:**	**22** **Up:**	**11** **Down:**	**9** **Up:**	**56** **Down:**	**112** **Up:**		**39** **Down:**
citT	glpD	manA	bdbB	acuA	abrB	fliM	cspB	gltC	acoA	abfA	ydzA	alsS
ctc	pyrE	manP	csfB	ald	ahpF	glyQ	dacA	hag	bdbA	acsA	yesL	cgeA
cydA	yckB	manR	dppC	amyE	aroE	hemC	feuA	ilvH	bdbB	amyE	yesM	cotV
cydB	yqjE	pyrC	fhuD	ansB	cspB	icd	flgE	purQ	csbA	araB	yesN	coxA
cydC		yjdF	gudB	bdbA	cspC	lytB	glpP	ydcS	csbC	araD	yfiD	cwlD
gtaB		yjdG	hbs	bofC	ctsR	mdh	pstC	yhfC	csbD	citM	yfiE	leuB
gutR		ynaF	lrpB	dctP	efp	oppD	yaaN	ykoL	dra	csbX	yfiH	leuS
icd		yydJ	nagP	dhaS	fabF	pdhC	yddT	yomL	etfA	cstA	yfmN	nin
ldh			spo0M	drm	frr	pgi	ydfF	ywnJ	etfB	ctaD	ygaO	opuE
mcsA			sunA	etfB	ftsH	pgk	ydjM		galT	dctB	yhaS	pta
mcsB			ugtP	hemC	gltP	pgm	yybN		glpF	dctR	yhaX	sigE
mrgA			ycnI	katE	leuD	rplE			gntP	fabHB	yhfW	spoIIIAC
mrpB			ycnK	kdgK	maeN	rplF			gspA	fbaB	yisK	spoVAA
oppA			yeeG	kdgT	mntH	rpsK			kipR	glpT	yjdB	ssb
phrK			yezE	mmsA	rplQ	rpsS			melA	hutH	ykuN	ybcD
smf			yfhC	narG	rpmB	tpiA			mleA	hxlA	ykzA	ybcH
spo0E			yfmQ	nupC	rpmF	yaaD			mleN	iolC	ykzE	ybcM
treP			yhdN	punA	rpmGB	ykrS			nagP	iolH	yocF	yckE
xpaC			yisQ	qcrA	rpsO	ykrT			ndk	iolI	yocG	ycxC
ydaD			yjbD	qoxB	rpsT	ylbQ			nhaX	kbl	yomT	ydfR
ydaS			yjeA	recO	smpB	yvfV			nupC	kdgA	yomZ	ydjE
ydaT			yjmF	resA	sspE	yvzB			odhA	kdgK	yonE	yesS
ydbD			ylqC	resB	yabR				odhB	kdgT	yonH	yezD
ydhK			yodC	resC	ybaR				pdp	lacA	yonK	yfhC
yebE			yoeB	resD	yccG				rpsC	lcfA	yopY	yhcA
yfhD			yozB	resE	yceJ				rsbV	malL	yoqF	yhcB
yfkJ			yxkO	rocC	yczH				rsbW	mtlR	yoqS	yhcC
yhzC				rplF	ydaH				rsbX	pel	yosB	yhjB
yjcG				sunT	ydgB				sdhA	phrC	yosD	yitJ
ykrS				ydhP	ydgF				sdhB	qcrB	yosJ	yjbD
yoqZ				yfiD	ydhB				sunT	qcrC	ypiF	yjeA
yoxC				yhfD	yebC				treR	rocB	ypzC	yocN
ytiA				yolJ	yfjT				ybyB	rocC	yqaD	yodC
ytzE				yoqX	yhbJ				ydaG	sacB	yqcK	yqaS
yuaF				ypwA	yhcB				ydhM	senS	yqkK	yqbM
yuaI				yraO	yjhB				ydhT	spoIIIAH	yqzG	yurO
yuiD				yrkA	ykoA				yfhD	sspO	yraO	yurP
yvaA				ysiA	ylaG				yflT	tyrZ	yrhP	yutK
ywfC				ytxE	yodJ				yhaP	xepA	ytcP	yvgW
ywmE				yuxI	yqeY				yolJ	xhlB	yteQ	
ywzA				ywiE	yqjL				yoxC	xkdH	ytlI	
yxiS				ywmA	yrvM				yqhA	xkdK	yugM	
yxzF					yuiF				yqhB	xkdN	yugN	
					yusG				ysbA	xkdO	yukJ	
					yutF				ysbB	xkdT	yulD	
					yuzG				ytiA	xkdV	yuxI	
					yvsH				ytxJ	xkdW	yvcA	
					yvzC				yvdI	xkdX	yvdJ	
					ywrK				yvdR	xsa	yveN	
									ywiE	xtmB	yvfL	
									ywjB	ycbO	yvfM	
									ywjC	ycbR	ywdC	
									ywqI	ycgB	ywmA	
									yxaI	ycsA	ywsA	
									yxiE	ydhN	yxeI	
									yxnA	ydhS	yxjC	

Up means: these genes are more abundant (i.e., less repressed) in the CcpA-mutant than in CcpA-wt. Down means: these genes are less abundant (i.e., more repressed) in the CcpA-mutant than in CcpA-wt. The numbers refer to Figures 2A,B and represent the number of uniquely up- or down-regulated genes per CcpA-mutant.

### The gene regulation of CcpA-T62H compared to wildtype CcpA

The CcpA-T62 is important for signal transduction from the HPr-Ser46-P interaction interface to the DNA binding domain (Schumacher et al., 2011). CcpA-T62H was found to be a stronger repressor. When the CcpA-T62H mutant strain was grown in LB + 1% glucose then only 9 genes were upregulated, but 50 genes were repressed (Table 5, Supplementary File, Sheets 1–3). Only 5 of these 59 genes were also found in the ΔCcpA strain, the other 54 genes did not have an altered regulation in the ΔCcpA strain (Figure 2A). The uniquely affected genes for CcpA-T62H are shown in Table 4. Genes from COG categories [C] Energy production and conversion, [G] Carbohydrate transport and metabolism, [J] Translation, ribosomal structure and biogenesis, and [P] Inorganic ion transport and metabolism showed a stronger regulation by CcpA-T62H (Figure 3). Only 3 of the 59 genes belonged to the CcpA regulon from SubtiWiki. When the CcpA-T62H strain was grown in LB without glucose then the regulatory effect of CcpA-T62H was even stronger than in LB + 1% glucose (Table 5, Supplementary File, Sheets 4–6). In LB without glucose there were 12 genes up- and 115 genes down-regulated (Figure 2B, Table 5, Supplementary File, Sheets 4, 5) and 55 of them belonged to the CcpA regulon from SubtiWiki. Genes from COG categories [C] Energy production and conversion, [G] Carbohydrate transport and metabolism, [K] Transcription, [M] Cell wall/membrane/envelope biogenesis, [R] General function prediction only, and [T] Signal transduction mechanisms showed a stronger regulation by CcpA-T62H (Figure 3).

**Table 5:** **The number of genes with altered regulation for each mutant compared to CcpA-wt as found in this transcriptomics study**.

	**LB + 1% glucose**		**LB**	
	**Up**	**Down**	**Up**	**Down**
ΔCcpA	189	27	55	34
CcpA-M17R	115	14	38	29
CcpA-T62H	9	50	12	115
CcpA-R304W	165	69	155	51

In total, 426 different genes had an altered regulation on LB + 1% glucose and 366 different genes had an altered regulation on LB without glucose (see also Figure 2).

### The gene regulation of CcpA-R304W compared to wildtype CcpA

The arginine on position 304 is important to contact the Ser46-P of HPr. We hypothesized that the CcpA-R304W had a reduced affinity for the glucose sensor protein HPr-Ser46-P but there is still a large number of affected genes (165 up and 69 down) when the strain was grown in LB + 1% glucose. When the CcpA-R304W strain was grown in the absence of glucose the number of affected genes was almost as high; there were 206 genes affected (155 up and 51 down) of which 151 genes were unique for CcpA-R304W (Figures 2A,B, Table 5 and Supplementary File, Sheets 4–6). Fifty nine of the affected genes in LB only belonged to the CcpA regulon from SubtiWiki. The overlap of CcpA-R304W with ΔCcpA is much larger in the presence of glucose (109 genes), than in the absence of glucose (32 genes, compare Figures 2A,B) showing that the HPr dependent gene regulation of CcpA is disturbed, but not the HPr independent gene regulation of CcpA. Therefore, we can conclude that the role of HPr-Ser46-P is less pronounced for this mutant.

### Functional analysis of the affected genes

For a more detailed look on the differences between the CcpA mutants all genes with a different regulation were functionally analyzed by categorizing those genes into Clusters of Orthologous Groups (COGs) (Figure 3). When the cells were grown on LB + 1% glucose then genes for energy production and conversion [C] were upregulated in all mutants, except for CcpA-T62H. Expression of genes for carbohydrate transport and metabolism [G] in ΔCcpA and CcpA-R304W showed the most difference. CcpA-T62H and CcpA-R304W showed a down regulation of translation, ribosomal structure and biogenesis genes [J] (Figure 3, LB + 1% glucose). When the cells were grown on LB without glucose then the CcpA-T62H and CcpA-R304W strains had most differences in gene regulation for genes in energy production and conversion [C] and carbohydrate transport and metabolism [G] compared to CcpA-wt (Figure 3).

### Growth of the strains with the CcpA mutants on C-medium

**Figure 4:**
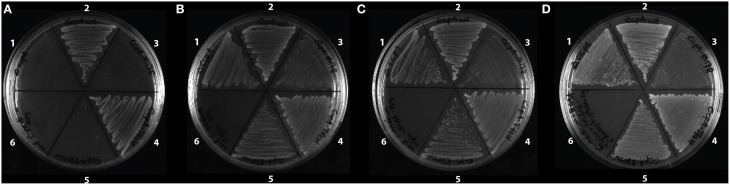
**Growth of the strains with the CcpA mutants (A) on C-medium, (B) on C-medium supplemented with glutamate, (C) on C-medium supplemented with glutamate and branched chain amino acids, and (D) on C-medium supplemented with glutamate, branched chain amino acids and uridine 5′-monophosphate**. All strains are *B.subtilis* ccpA::spec and strain 2-5 have ccpA on a plasmid. 1, ΔccpA; 2, ccpAwt; 3, ccpA-M17R; 4, ccpA-T62H; 5, ccpA-R304W; 6, empty.

A *ccpA* knockout strain shows a growth defect in glucose minimal medium (Faires et al., 1999; Fujita, 2009). Here we tested how the CcpA mutant strains performed in glucose minimal medium. In the strains with the CcpA mutants, some genes coding for proteins responsible for amino acid catabolism were deregulated. One of them was the gene *rocG* coding for glutamate dehydrogenase. Normally, the *rocG* gene is repressed by CcpA, but in both growth conditions used in this study and in strains with all CcpA variants except CcpA-T62H, the repression of *rocG* was released. Higher levels of RocG decrease the level of glutamate, which impairs the growth of these strains on a glucose minimal medium (Fujita, 2009). The altered gene regulation of *rocG* caused a growth deficiency of the CcpA mutant strains on minimal medium, which was shown on C-medium agar plates for the strains with the ΔCcpA, CcpA-M17R, and CcpA-R304W mutations (Figure 4A). The CcpA-T62H strain still repressed the *rocG* expression and did not have a growth defect (Figures 4A,B). The growth deficiency of the mutants was almost fully restored to the same extent as CcpA-wt when the C-medium agar plates were supplemented with glutamate, except for CcpA-M17R (Figure 4B) and fully restored when the C-medium agar plates were supplemented with glutamate and branched chain amino acids (BCAA), except for CcpA-M17R (Figure 4C). The growth defect of the CcpA-M17R mutant strain was partially restored by the addition of glutamate (Figure 4B); however the addition of only glutamate was not enough. The remaining growth defect of the CcpA-M17R strain might be explained by the *pyrE* gene. PyrE is responsible for pyrimidine biosynthesis (Subtiwiki, 2014) and its gene expression was only down-regulated in the CcpA-M17R strain. This strain did not show a growth defect in LB because LB is rich in nucleotides from the yeast extract. Therefore the C-medium was also supplemented with uridine 5′-monophosphate in addition to glutamate and BCAA, but the growth defect of the CcpA-M17R mutant strain was not restored (Figure 4D). The residual growth deficiency remains elusive and is probably dependent on more additives.

### Examination of cre sites for analysis of CcpA-M17R

The methionine on position 17 in CcpA is involved in DNA binding, therefore the sequence of the *cre* site could play a role in the gene regulation by CcpA-M17R. Unfortunately, not all genes affected by CcpA-M17R could be taken into account, because many genes have no *cre* site; they are subjected to indirect control by CcpA (Ludwig et al., 2002; Görke and Stülke, 2008; Marciniak et al., 2012). All known *cre* sites for genes that had an altered regulation in the CcpA-M17R mutant were compared. Genes were grouped into three classes for the analysis; only affected in the ΔCcpA strain, affected in both ΔCcpA and CcpA-M17R, and only affected in the CcpA-M17R strain. The *cre* sequences were analyzed for their palindromic nature, but the differences between the groups of *cre* sites were not clear enough to draw conclusions (see Supplementary File, Sheet 8, Tables 1, 4). Furthermore, the similarity of each group of *cre* sites to the consensus *cre* site was compared but there was no significant difference between all three regulatory groups (see Supplementary File, Sheet 8, Tables 1, 4).

Weblogos for the groups of *cre* sites affected only in CcpA-M17R showed that these *cre* sites had a higher occurrence of G on position 4 and a less defined nucleotide on position 10 (Supplementary File, Sheet 8, Table 1). The methionine on position 17 was shown to bind to thymine12 in the *ackA cre* site (Schumacher et al., 2011), and the less defined nucleotide on position 10 found here could mean that the arginine reached further into its surroundings than the methionine.

The consensus *cre* site is W_1_ T_2_ G_3_ N_4_ N_5_ A_6_ R_7_ C_8_ G_9_ N_10_ W_11_ W_12_ W_13_ C_14_ A_15_ W_16_ and the G_3_, C_8_, G_9_, C_14_ are most conserved (Schumacher et al., 2011). Seven of the thirteen genes or operons with a known *cre* site that were affected by CcpA-M17R also had a T on position 12 in the *cre* site, the other six had a C (3x), a G (2x), or an A (1x) on position 12, but those six had a T on position 11 (5x) or position 13 (1x). This finding could mean that the arginine on position 17 in CcpA also contacted a thymine in the *cre* site and that the longer sidechain of arginine could reach further into the surrounding (Supplementary File, Sheet 8, Tables 1, 4).

### Sensitivity for extracellular glucose

Earlier work shows that the transport of glucose decreases in a CcpA knockout strain (Ludwig et al., 2002). We hypothesize that a decreased glucose uptake would result in a decreased growth rate. The growth rates of the different CcpA strains were examined on LB + 1% glucose and the ΔCcpA, CcpA-M17R, and CcpA-R304W strains grew a little bit slower than the CcpA-wt strain (Supplementary File, Sheet 12), which could be due to a decreased glucose uptake. The growth of the CcpA-T62H strain was the same as the CcpA-wt strain, which could be explained by the strong repressive mode of CcpA-T62H.

None of the *pts* genes, the glucose permease, or glucose symporter were differently expressed in LB + 1% glucose or in LB in any of the mutant strains, which means that the uptake of glucose was not regulated at the level of gene expression, as shown before (Singh et al., 2008).

## Discussion

CcpA is a global regulator of carbon catabolite control in *B. subtilis* and needs HPr or Crh as a coeffector (Moreno et al., 2001; Sonenshein, 2007; Fujita, 2009). It has been previously estimated that there are 150 *cre* sites in *B. subtilis*, which are involved in the regulation of around 300 genes (Fujita, 2009). Moreno *et al.* reported in a genome-wide transcriptome study that about 85 genes are activated by CcpA and 250 genes are repressed by CcpA in LB + glucose medium (Moreno et al., 2001). However, according to the CcpA regulon from SubtiWiki (Mäder et al., 2012; Subtiwiki, 2014) there are only 11 genes activated by CcpA and 202 repressed. The genes mentioned in the SubtiWiki CcpA regulon are probably directly regulated by CcpA; the class 1 genes (Ludwig et al., 2002) and the other genes found by Moreno et al. are probably indirectly regulated; the class 2 genes (Ludwig et al., 2002). Class 1 genes have a *cre* site and class 2 genes are affected when CcpA alters the glucose uptake (through altered phosphorylation of HPr) and thereby changes the concentration of intracellular inducers (Ludwig et al., 2002; Blencke et al., 2003). There are also class 0 genes, which are independent of CcpA (Ludwig et al., 2002). In a transcriptome study Yoshida et al. have found 66 genes of which the repression was glucose dependent (Yoshida et al., 2001). Here we found 244 genes which had a glucose dependent regulation in one of the CcpA mutant strains (Supplementary File, Sheet 2).

Marciniak et al. (2012) have categorized all known (and predicted) *B. subtilis cre* sites from high to low affinity. To do so, they have replaced the native *ccpA* promoter by an inducible promoter, then the fold change in gene expression was examined for a low, medium and high level of *ccpA* expression. The correlation between the fold change in gene expression and the position of the *cre* site compared to the transcriptional start site (TSS) was mapped (Marciniak et al., 2012). The information found was used in this transcriptomics study to sort the affected genes on the basis of *cre* to TSS distance, and check whether it correlated to the fold change in gene expression but there was no clear correlation between fold change and *cre* to TSS distance. The fold change was very similar over a range of *cre* to TSS distances (Supplementary File, Sheet 7).

The genes that are controlled by CcpA are in most cases also controlled by a second regulator, which is substrate specific (personal communication with Jörg Stülke). Thus, it is impossible to find the whole CcpA regulon in the ΔCcpA experiment. The other genes found in this study that do not belong to SubtiWiki's CcpA regulon could be unidentified members of the CcpA regulon, or a member of the class 2 genes (Ludwig et al., 2002). On the other hand, the CcpA regulon from SubtiWiki contains 213 genes, but 152 of them were not altered in the ΔCcpA strain. One explanation for the missing genes from the CcpA regulon from SubtiWiki could be that the list of genes in the CcpA regulon from SubtiWiki is composed from various experiments with various growth conditions, e.g. also from late exponential, or stationary growth phase or in minimal media containing glucose.

18 genes from the CcpA regulon from SubtiWiki were affected both in the presence and absence of glucose; the *rbsRKDACB* operon for ribose was strongly deregulated in LB + 1% glucose and the *licHA, bglHP*, and *gntK* are slightly deregulated in the presence of glucose and the other seven genes were the same in both conditions (Supplementary File, Sheet 11).

The changes in gene regulation in absence of glucose in the ΔCcpA strain also showed that CcpA was involved in glucose independent gene regulation, as has been shown before (Moreno et al., 2001). Moreno et al. show that those genes are not involved in carbon metabolism (Moreno et al., 2001), but that was not the case in this study. 46 of these 89 genes could be grouped into only four COG-categories: Energy production and conversion [C], Amino acid transport and metabolism [E], Carbohydrate transport and metabolism [G], and Transcription [K] (Figure 3 and Supplementary File, Sheet 10).

The neutral methionine on position 17 in CcpA was changed to a positively charged arginine. The M17R mutation was expected to alter the DNA-binding affinity of CcpA; the positively charged arginine could cause stronger binding to the negatively charged DNA. CcpA-M17R is probably a weaker repressor, because the number of differentially regulated genes was lower than in the ΔCcpA strain. All upregulated genes are most likely the result of a weaker repression by CcpA-M17R because CcpA is mainly involved in gene repression. However, it was hard to explain the altered gene regulation by effects on DNA binding, as shown by *cre* site comparison; there were no clear differences in the *cre* sites from genes that were only affected in the ΔCcpA strain or in the CcpA-M17R strain (Supplementary File, Sheet 8, Tables 1, 4). The palindromicity of the *cre* sites in the group of genes that was affected by CcpA-M17R was lower than in the group of genes that was only affected by ΔCcpA; this could indicate that the palindromicity of the *cre* site is less important for CcpA-M17R.

The CcpA protein consists of a N-terminal DNA binding domain and a core domain for coeffector and HPr binding, linked together with a hinge helix region. The threonine on position 62 is located in the core protein, just above the hinge-helix, and is important for the conformational change in the CcpA protein upon HPr binding (Schumacher et al., 2011). This conformational change is believed to improve DNA binding. The threonine to histidine mutation on position 62 has been found during a random mutagenesis study of CcpA-T62, where CcpA-T62H was found to repress *xynP* strongly in the absence of glucose (Thesis G. Seidel, Erlangen, Germany, 2005).

We found a large number of down-regulated genes in the CcpA-T62H strain, especially in the absence of glucose. Therefore, we suggest that the CcpA-T62H variant was a stronger repressor than CcpA-wt. The downregulated genes might not be regulated by wildtype CcpA under the chosen conditions. The high number of affected genes in LB without glucose could indicate that the role of HPr-Ser46-P for complex formation was less important for this mutant, since catabolite repression was observed in the absence of glucose. There were only a few genes upregulated in case of CcpA-T62H compared to CcpA-wt and the other mutants. We can conclude that the T62H substitution forces CcpA into a conformational change that happens normally in CcpA upon HPr-Ser46-P binding; this change is probably beneficial for DNA binding. The binding of a CcpA mutant protein to *cre* sites without the prior binding of CcpA to the corepressor HPr-Ser46-P was observed before for five CcpA mutants in *B. megaterium* (Kuster-Schock et al., 1999).

The most important residue for the interaction of CcpA with HPr-Ser46-P is the arginine at position 304 in CcpA (Sprehe et al., 2007; Schumacher et al., 2011). HPr will be phosphorylated on serine 46 by HPrK/P when glucose is used as a carbon source (Fujita et al., 1995; Görke and Stülke, 2008) and subsequently HPr-Ser46-P will form a complex with CcpA. The formation of this complex stimulates the CcpA-HPr-Ser46-P heterodimer formation needed to bind to DNA (Deutscher et al., 1995; Fujita et al., 1995; Schumacher et al., 2011). The positively charged arginine at position 304 was mutated to the neutral, but bulkier tryptophan which probably made binding of this CcpA mutant to the negatively charged HPr-Ser46-P more difficult.

The large number of genes affected by CcpA-R304W in the presence of glucose suggest HPr independent gene regulation. There were 234 genes affected by CcpA-R304W in the presence of glucose, 205 genes were affected in the absence of glucose, and 69 genes were affected in both conditions. The *rbsRKDACB* operon for ribose was strongly deregulated in the ΔCcpA strain, but much less deregulated in CcpA-R304W (Supplementary File, Sheet 11) indicating that the repressor function of CcpA-R304W decreased which is probably due to the decreased binding of HPr-Ser46-P. The large number of genes affected by CcpA-R304W in the absence of glucose also suggest HPr independent gene regulation, because HPr is not phosphorylated in the absence of glucose so the CcpA-HPr complex formation is reduced but CcpA-R304W is still affecting the regulation of many genes (Table 5, Supplementary File, Sheets 4, 5).

Another explanation could be that the signal transduction in the protein has changed due to the mutation. Maybe the R304W mutation fixes CcpA into the HPr-Ser46-P bound conformation. Or possibly binding of the coeffectors fructose-1,6-bisphosphate (FBP) and glucose-6-phosphate (G6P) is more difficult. Earlier work shows that the affinity of HPr-Ser46-P to CcpA increased more than twofold by the presence of 40 mM FBP or G6P (Seidel et al., 2005), but if coeffector binding is reduced then the affinity of CcpA-R304W to HPr-Ser46-P is also reduced. It will be interesting to measure the binding affinity of CcpA-R304W to HPr-Ser46-P with SPR in a future study.

It is known that a ptsH-S46A strain lost part of its repressing power (Deutscher et al., 1994; Martin-Verstraete et al., 1995) and here we showed that CcpA-R304W also lost part of its ability to repress gene expression. These results show that CcpA and HPr depend on each other and either one of the mutations makes complex formation more difficult.

The regulation of genes in some COGs really differed from CcpA-wt, i.e., they were not regulated by the CcpA-wt but only by the CcpA-mutant. Genes in COGs for Energy production [C], carbohydrate metabolism [G], translation [J], posttranslational modification [O], and inorganic ion transport [P] are more strongly repressed in the CcpA-T62H and CcpA-R304W mutants than in the CcpA-wt strain in LB + 1% glucose (Figure 3) which shows the differential regulatory character of the CcpA mutants.

In general, we should take into account that the CcpA mutants studied here could have effects on gene regulation which cannot be seen in LB medium with a CcpA mutant because there might be unknown triggers for CcpA which are absent in LB ± glucose. Furthermore, LB medium without added glucose still contains low amounts of glucose and other repressing sugars which might still control the most sensitive target genes. The mutations might uncouple regulation from these triggers. Future experiments in chemically defined medium or in a HPr knockout in the CcpA mutant strains could be done to reveal triggers involved in CcpA dependent gene regulation which were not present when the strains were grown in LB.

In conclusion, the point mutations in CcpA in this study were made to get more insight into the gene regulation mechanisms of CcpA. The main finding here was that the M17R mutation resulted in a small relief of CCR. CcpA-T62H was a stronger repressor than CcpA-wt and can do CCR also in the absence of glucose. CcpA-R304W had a strong regulatory affect in presence and absence of glucose and we suggest that CcpA-R304W is less dependent on HPr-Ser46-P, FBP, and G6P. However, SPR measurements should show the binding affinity of the CcpA mutants to the *cre* sites in presence or absence of HPr-Ser46-P.

## Funding

RD and OK were financially supported by a specific SYSMO2 grant from ALW-NWO. GS was financially supported by a specific SYSMO2 grant from the BMBF.

### Conflict of interest statement

The authors declare that the research was conducted in the absence of any commercial or financial relationships that could be construed as a potential conflict of interest.

## Supplementary material

Supplementary File **CcpAmut sup_INFO_1.xlsx**. The titles of the 12 different sheets in the file are listed below. Row 1 in every sheet gives an explanation of the data shown in that sheet. 1_LBgluc_All signif_fold>1.7; 2_LBgluc_Venn_diagram_fold>1.7; 3_LBgluc_Regulatory_groups; 4_LB_ALLsignif_fold>1.7; 5_LB_Venn_diagram_fold>1.7; 6_LB_Regulatory_groups; 7_LBgluc_CRE-TSSdistance; 8_cre_palindromicity; 9_LBgluc_COGcat; 10_LB_COGcat; 11_CcpA_regulon; 12_growth curves.
